# First Case Report of Smith–Magenis Syndrome (SMS) Among the Arab Community in Nazareth: View and Overview

**DOI:** 10.1097/MD.0000000000002362

**Published:** 2016-01-22

**Authors:** Yousif Nijim, Amin Adawi, Bishara Bisharat, Abdalla Bowirrat

**Affiliations:** From the Head of Pediatric and Neonatal Department (YN); Pediatric and Neonatal Department, EMMS Nazareth—The Nazareth Hospital, Nazareth (AA); Director of EMMS Nazareth Hospital, Galilee Medical School – Bar Ilan University (BB); and Clinical Neuroscience and Population Genetics—EMMS, Nazareth Hospital, Nazareth, Israel (AB).

## Abstract

Smith–Magenis syndrome (SMS0) is a complex and rare genetic multisystem disorder characterized by a variable pattern of cognitive deficits accompanied by a1 distinctive behavioral phenotype. SMS is characterized by subtle facial dysmorphology, short stature, sleep disturbances, and neurobehavioral abnormalities. Little is known about the manifestation of his unique case among Arab population and its strategic treatment.

This study comes to present a case of SMS in an Arab newborn male who was born in spontaneous delivery on June 29, 2015, with tachypnea, tracheomalacia, and mild hypotonia. The newborn was admitted on the Neonatal Intensive Care Unit (NICU), and various laboratory examinations and clinical examinations were performed.

Throughout his hospitalization, feeding difficulties appeared and thus a peripheral venous catheter was inserted in the left leg.

After 22 days of follow-up and hospitalizations, the patient status improved and he was discharged with recommendations to be in follow up in pediatric outpatient clinic.

However, notwithstanding the different investigations, intermittent tachypnea continued at a rate of 72 to 77 breaths/min. Search for diagnosis begin intensively owing to persistence of tachypnia, mild hypotonia, feeding difficulties, sleep disturbances, and mild dysmorphic facial features. Suspicions of genetic abnormalities were considered and blood samples were sent for chromosome analysis and for fluorescent in situ hybridization (FISH) testing.

The genetic results revealed the following: cytogenetic findings: 46, XY, del(17)(p11.2) and the FISH results: del(17)(p11.2p11.2) (D17S29). The chromosome diagnosis revealed an interstitial deletion of 17p11.2 and the diagnosis of the SMS was confirmed.

Accurate clinical diagnosis, therapeutic assessments and a holistic management plans, including multidiscipline therapeutic strategies, periodic neuro-developmental assessments, and an early intervention programs, are recommended.

However, cytogenetic analysis or FISH using an *RAI1*-specific probe is the most frequently used technique for DS. Sleep and behavioral disturbances treatment include a combination of the daytime dose of acebutolol with an evening oral dose of melatonin. Melatonin as chronobiotic, antioxidant, and analgesic agent showed to be effective in different primary sleep disorders and in those associated with neurobehavioral disorders. Based on the beneficial effect of melatonin, it will be useful to use serum levels of melatonin as a follow-up test.

## INTRODUCTION

Smith–Magenis syndrome (SMS, OMIM 182290) is a complex genetic multisystem disorder characterized by a variable pattern of cognitive deficits accompanied by a distinctive behavioral phenotype that includes a range of challenging behaviors, including self-injury, aggression, impulsivity, attention seeking, and sleep disturbance in addition to developmental delay.^1,2^

SMS is still not well-known because it is an extremely rare misdiagnosed disorder characterized by subtle facial dysmorphology that progresses with age and include brachycephaly, mid-facial hypoplasia with broad flat midface, broad nasal bridge, prognathism, and clinical features that overlap with other developmental delay such as infantile hypotonia, short stature, and intellectual disability syndromes as 1p36 deletion syndrome, 9q34 deletion syndrome (Kleefstra syndrome), 22q11.2 deletion syndrome (DiGeorge syndrome or Velocardiofacial syndrome), Fragile x syndrome, brachydactyly-intellectual deficit syndrome (del 2q37), Down syndrome, Prader–Willi syndrome, Sotos syndrome, and Williams syndrome. Additionally, SMS may be misdiagnosed as autism, ADHD, obsessive-compulsive disorder, or oppositional defiant disorder.^3,4^

Cytogenetic analysis using Comparative Genomic Hybridization (Acgh) or fluorescent in situ hybridization (FISH-specific probes for SMS) is warranted in individuals suspected of having SMS who had a prior “normal” chromosome analysis.

The features of SMS are likely to range in severity from person to person (variable expressivity). SMS is an unpredictable and randomly occurring condition that has no identifiable risk factors; it occurs in roughly 1 in 15,000 to 25,000 births and was first described by Ann C. M. Smith and her colleagues in 1982.^5^

The inheritance pattern of SMS is autosomal dominant, but most cases are sporadic, although parental mosaicism and rare heritable chromosome rearrangements that lead to this complex contiguous gene syndrome is associated with an interstitial microdeletion at the 17p11.2 chromosome region that includes the retinoic acid-induced 1 (RAI1) gene (around 90% of cases) or by RAI1 gene point mutation (10% of cases of SMS).^6–8^

The gene functions of RAI1 are linked to regulation of several genes that control specific pathways of various biological processes.

Haploinsufficiency of *RAI1* gene results in SMS and may be one of the early signs that an individual might have this disorder.^9,10^

In conjunction with neuropsychiatric, various congenital abnormalities, a disrupted circadian sleep-wake pattern (light/dark cycle) is a trademark of SMS. Previous studies reveal that >75% of all patients with SMS present with significant sleep disturbance, and an inverted circadian rhythm of plasma melatonin, urinary melatonin, and urinary 6-sulfatoxymelatonin.

Physiologically, circadian rhythm located in the supra-chiasmatic nucleus within the hypothalamus and has plethora of functions, in addition to its critical regulator task of sleep-wake cycle, it is responsible of body temperature maintenance, glucose homeostasis, feeding cycles, cell-cycle progression, hormone secretion, and drug metabolism.^11^

An aberrant rhythm of melatonin (*N*-acetyl-5-methoxytryptamine) secretion is considered as the underlying reason of sleep disturbance, providing evidence that these circadian-rhythm abnormalities can also have a great impact not only on sleep pattern but also on disease vulnerability, severe behavioral phenotype, cognition impairment, and loss of learning capabilities.^12–15^

This notion strengthens the hypothesis that haploinsufficiency of the circadian system gene may be responsible for sleep disturbances,^3^ and *RAI1* may play a fundamental molecular role in the sleep cycle and in the core molecular pathway of circadian rhythm.^16^

This hypothesis condemning *RAI1* mutation directly in circadian function,^17^ even though the exceptional cases of 17p11.2-deletion, in individuals who have atypical large deletion and normal melatonin secretion, but they still have sleep disturbance.^16^

Indeed, the *RAI1* might play a crucial role in the regulation of gene transcription and circadian maintenance. Examinations into the task that *RAI1* plays in the regulation of gene transcription and circadian maintenance revealed that *RAI1* plays a positive transcriptional regulator of circadian locomotor output cycles kaput (*CLOCK)*, a vital constituent of the mammalian circadian oscillator that transcriptionally regulates numerous crucial circadian genes.^18–20^

Given the phenotypic consequences of RAI1 mutation or deletion, RAI1 impacts are uncountable and are reflected negatively in pathways associated with developmental, neuropsychiatric function, and circadian rhythm.^21^

Understanding and detection of the molecular defect that the result from *RAI1* mutation or deletion and the abnormality of circadian-gene expression and the inverted melatonin secretion observed in almost all patients is almost certainly a consequence of this disorder. Thus, *RAI1* acts directly or through multiple channels to influence or as a regulator of *CLOCK,* might have multiple consequences on different pathways as a result of RAI1 haploinsufficiency, and gives the opportunity for pharmaceutical, behavioral, or nutritional-based interventions.

The diagnosis of SMS is based on clinical findings which include the involvement of multisystem disorders characterized by multiple congenital anomalies accompanied with physical, cognitive, and behavioral malfunctions and confirmed by either detection of an interstitial deletion of 17p11.2 or by molecular genetic testing of *RAI1*.^7,22^

Typically this disorder is diagnosed by detection of an interstitial deletion of 17p11.2 (ranging from 1.5 to 9 Mb) by G-banded cytogenetic analysis and/or by FISH using a DNA probe that contains *RAI1* or D17S258 and the deletion is observed in 90% to 95% of cases. However fluorescent *in situ* hybridization (FISH) using an *RAI1*-specific probe is the most frequently used technique.^4,23^

Furthermore, sequence analysis and deletion/duplication analysis for the *RAI1* gene can also be used for detection of SMS in 5% to 10% and 95% of cases, respectively. Micro array CGH tests may also detect SMS. Although the use of Multiplex ligation-dependent probe amplification (MLPA) and real-time qPCR for a rapid, cost-effective diagnosis is already underway in many research laboratories in Europe, and most clinical and diagnostic labs in the United States are yet to adopt these technologies.

Pathogenic variants detected by sequence analysis may include small intragenic deletions/insertions and missense, nonsense, and splice site variants; typically, exon or whole-gene deletions/duplications are not detected. Sequence analysis (particularly of exon 3, in which all pathogenic variants have been found to date) detects *RAI1* pathogenic variants in individuals with SMS when cytogenetic and FISH studies are negative for the 17p11.2 deletion.^4,24^

To confirm the diagnosis in proband, different steps are needed: array CGH should be performed as an initial study. This test will identify all 17p11.2 deletions and will also identify phenotypically overlapping genomic disorders. If there is a strong clinical suspicion of SMS and aCGH is normal, deletion/duplication analysis specific for *RAI1* may be performed. If the above deletion analyses are normal, sequencing of *RAI1* should be considered. Prenatal diagnosis and preimplantation genetic diagnosis (PGD) for at-risk pregnancies require prior identification of the pathogenic deletion or allelic variant in the family.

As for the management of this unique disorder whose diagnosis is subtle, owing to multisystem involvement and variable presentations of SMS, a various clinical and diagnostic evaluation should be carried out for effective management. As its symptoms are interwoven with a plethora of other developmental disorders and intellectual disability syndromes, making the pursuit for a magic cure is challenging. The therapeutic management of the sleep disorder in SMS remains a challenge for physicians and parents and no specific ideal treatment is available yet and no single regimen shows consistent efficacy, even though, different polypharmacy and/or serial trials are being tested.^25^

Nevertheless, a holistic management plan for people with SMS including multidiscipline therapeutic strategies, periodic neurodevelopmental assessments/or developmental/behavioral pediatric consultations, and an early intervention programs are warranted to tackle the various symptomatologies of SMS.

Indeed, beside the medical treatment, special education such as, vocational training/supports later in life, speech/language intervention, physical, occupational, behavioral, and sensory integration therapies are needed.

However, different psychotropic drugs have been used to alleviate the various symptomatologies of SMS. These medications include stimulants, antidepressants, antipsychotics, hypnotics, mood stabilizers, and alpha 2 agonists. In spite of the severe collateral effects of antipsychotics, Risperidon at a dosage <4 mg is reported not to have side effects and may reduce aggression and impulsivity significantly in children and adolescents.^6,8,26–29^

Affected individuals may also benefit from use of psychotropic medication to increase attention and/or decrease hyperactivity, and sleep disorders. Respite care and psychosocial support for family members are recommended.

Pharmacologic intervention should be considered on an individual basis with recognition that some medications may exacerbate sleep or behavioral problems and may cause weight gain. For example, a patient with SMS was documented to have a serious adverse event taking Strattera^®^ (Atomoxetine Hydrochloride) with extreme escalation of behaviors and aggression leading to hospitalization. Benzodiazepines are also contraindicated and have very low efficacy, suggesting that use of these drugs may be detrimental to individuals with SMS.^25^

Furthermore, sleep disturbance is an almost universal finding and a cardinal feature of SMS and is considered to be among the most difficult to handle because lack of sleep appears to exacerbate other psychiatric symptoms in people with SMS.

Melatonin that functions as chronobiotic, antioxidant, and analgesic agent has been shown to be effective and beneficial in different primary sleep disorders and also in those associated with neurobehavioral disorders. Serum melatonin levels in patients with SMS have been shown to be abnormally low at night (when they should be high) and relatively high during the day. De Leersnyder et al (2001) and Potocki et al (2000)^14,15^ examined 27 SMS patients in whom 24-h melatonin was analyzed; the results show that in 26 patients (96%) a diurnal melatonin rhythm shifted by ∼12 h from the normal pattern of secretion.^14,15^

Early anecdotal reports of therapeutic benefit from melatonin at a dose low (≤3 mg) taken at bedtime remain encouraging, providing variable improvement of sleep without reports of major adverse effects. De Leersnvder et al^14^ reported uncontrolled study of 9 patients with SMS treated with oral ß-1-adrenergic antagonists (acebutolol 10 mg/kg/day). The results showed suppression of daytime melatonin peaks and subjectively improved behavior, but the treatment did not restore nocturnal plasma concentration of melatonin. Other uncontrolled study reported by the same authors^20^ combined the daytime dose of acebutolol (10 mg/kg administered at 0800 h) with an evening oral dose of melatonin (6 mg at 8 pm) and they found that nocturnal plasma concentration of melatonin was restored and nighttime sleep improved with disappearance of nocturnal awakenings. It is worth to mention that contraindications to the use of ß-1-adrenergic antagonists include asthma, pulmonary problems, some cardiovascular disease, and diabetes mellitus.^12,20^

Based on the beneficial effect of melatonin, it will be useful to use serum levels of melatonin as a follow-up test. In fact, measuring serum levels of melatonin among SMS cases whose deletion size is common and has been specified as typical deletion may be an ideal for these patients. In contrast, the limitation of this test may lie in patients with large, atypical distal 5 Mb deletion of paternal origin, who exhibit normal melatonin rhythm, and then the melatonin test role in these cases is useless.

## CASE REPORT

We report a case study of a newborn male who was born in spontaneous delivery at term on June 29, 2015, to a 27 years old mother with gestational diabetes A1. Apgar score at 1 min after birth was 9 and at 5 min after birth was 10. Birth weight was 3142gr, head circumference was 35 cm, length = 47 cm, pulse = 118, breathing rate = 72/minute, blood pressure: systolic = 69, diastolic = 34, blood saturation=100.

The newborn was admitted to the Neonatal Intensive Care Unit (NICU) at the age of 2 days due to tachypnea (up to 88 breaths/min), tracheomalacia, and mild hypotonia. During his admission at the NICU various laboratory examinations were conducted including CBC, electrolytes, biochemistry, thyroid function tests, and 2 blood cultures, which were all normal.

Clinical examinations for whole body systems were performed: chest x-rays, EEG, cranial ultrasound, and heart echocardiogram, which were all intact. Moreover, the neurological examination was normal: the plantar grasp reflex, Moro reflex, and sucking reflex were all normal expect mild hypotonia. During his hospitalization, feeding difficulties appeared and thus a peripheral venous catheter was inserted in the left leg. Further metabolic investigations were performed including blood ammonia, Lactate, blood amino acids and organic acids, and finally cerebrospinal fluid (CSF) amino acids. The results of all these investigations were normal. After 22 days of follow-up and hospitalizations, the patient status improved and he was discharged with recommendations to be in follow-up in pediatric outpatient clinic.

**FIGURE 1:**
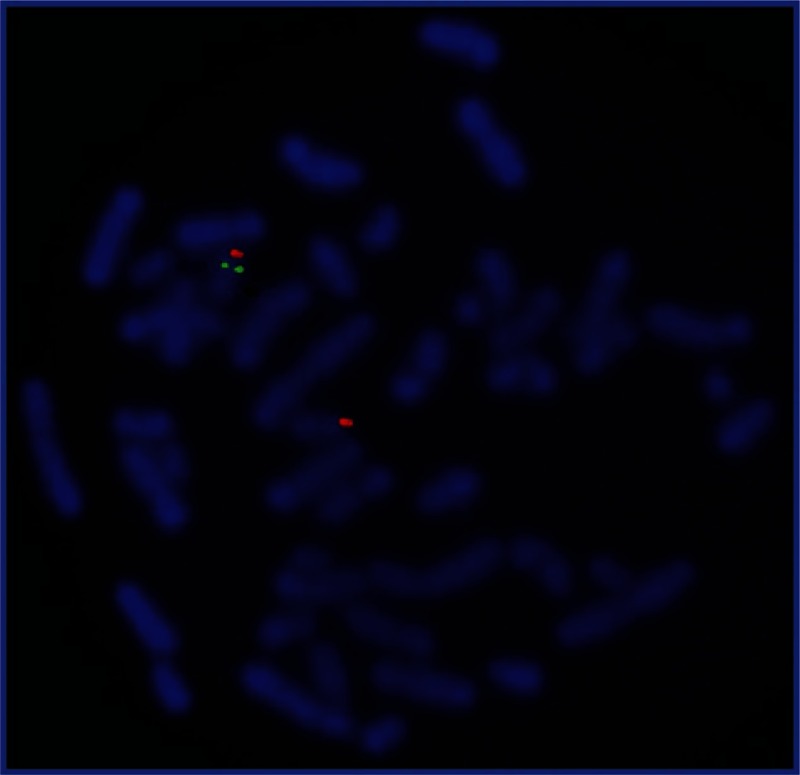
Metaphase fluorescent in situ hybridization (FISH) using the probe in the Smith–Magenis syndrome (SMS, OMIM 182290). The red area shows the control the green gene responsible for SMS. FISH = fluorescent in situ hybridization, SMS = Smith–Magenis syndrome.

However, notwithstanding all these investigations, intermittent tachypnea continued at a rate of 72 to 77 breaths/min. Owing to persistence of tachypnia, mild hypotonia, feeding difficulties, sleep disturbances, and mild dysmorphic facial features observed clearly such as a flat, broad nasal bridge and small mandible, suspicion of genetic abnormalities were considered and blood samples were sent for chromosome analysis and for FISH testing (Figure 1). The genetic exam revealed the following results:

Cytogenetic findings: 46,XY, del(17)(p11.2) and the fluorescence in situ hybridization results: del(17)(p11.2p11.2) (D17S29). The chromosome diagnosis revealed an interstitial deletion of 17p11.2 and the diagnosis of the SMS was confirmed.

Today, the child is in follow-up every 3 months in a pediatric outpatient clinic. His overall medical condition is good, without feeding problems, he is gaining enough weight and he has normal breathing rate (35 breaths/min). As for the sleep disturbances symptoms, a combination of the daytime dose of acebutolol (acebutolol 10 mg/kg/day) with an evening oral dose of melatonin (3 mg at 8 pm) was given to this child and he improves wonderfully. Currently, no other medications prescribed to him.

## DISCUSSION

SMS is a multisystem genetic disorder characterized by multiple congenital anomalies accompanied with physical, cognitive, and behavioral malfunctions. The underpinning causes of this rare disorder are a heterozygous interstitial microdeletion involving chromosome 17p11.2, where the retinoic acid-induced 1 (*RAI1*) gene is located, or by a point mutation of *RAI1*, causing haploinsufficiency of the gene. Functional assays have identified collection of genes that are either upregulated or downregulated as a consequence of *RAI1* haploinsufficiency.^1,2^

One of the cardinal signs of SMS is severe sleep disturbances and maladaptative daytime behavior (ie frequent nighttime awakenings, sleepiness in day/frequent napping, early morning awake times). These features have been linked to an abnormal circadian secretion pattern of melatonin. This irregular diurnal (inverted) circadian rhythm of melatonin appears pathognomic in SMS.^30–31^

Physiologically, circadian rhythm located in the supra-chiasmatic nucleus within the hypothalamus and has plethora of functions; in addition to its critical regulator task of sleep-wake cycle, it is responsible of body temperature maintenance, glucose homeostasis, feeding cycles, cell-cycle progression, hormone secretion, and drug metabolism.

An aberrant rhythm of melatonin secretion is considered as the underlying reason of sleep disturbance, providing evidence that these circadian-rhythm abnormalities can also have a great impact not only on sleep pattern but also on disease vulnerability, severe behavioral phenotype, cognition impairment, and loss of learning capabilities.^12–15^

This notion strengthens the hypothesis that haploinsufficiency of the circadian system gene may be responsible for sleep disturbances,^3^ and *RAI1* may play a fundamental molecular role in the sleep cycle and in the core molecular pathway of circadian rhythm.^16,24^

The diagnosis of SMS is based on clinical findings which include the involvement of multisystem disorders characterized by multiple congenital anomalies accompanied with physical, cognitive, and behavioral malfunctions, confirmed by multiple genetic testing. The clinical suspicion of SMS must be established by detection of micrdeletion of 17p11.2 using G-banded cytogenetic analysis and/or by FISH using a DNA probe or by targeted aCGH. In 95% of cases microdeletion is detected and diagnosis of SMS is confirmed. In other cases where microdeletion is absent, sequence analysis for the *RAI1* gene can be used for detection of mutations. If no mutation was found, whole genome aCGH analysis should be considered.^4,24^

Concerning the therapeutic intervention of SMS, no treatment is currently available and no single regimen shows consistent efficacy;^25^ nonetheless, different psychotropic drugs have been used to alleviate the various symptomatologies of SMS. These medications include stimulants antidepressants, antipsychotics, hypnotics, mood stabilizers, and alpha 2 agonists. Risperidon at a dosage <4 mg is reported not to have side effects and may reduce aggression and impulsivity significantly in children and adolescents.

As for the sleep disturbances symptoms, various medications have been shown beneficial in different primary sleep disorders and also in those associated with neurobehavioral disorders, these medications include melatonin at a low dose (≤3 mg) taken at bedtime, oral ß-1-adrenergic antagonists (acebutolol 10 mg/kg), or a combination of the daytime dose of acebutolol with an evening oral dose of melatonin (6 mg at 8 pm).

Based on the beneficial effect of melatonin, it will be useful to use serum levels of melatonin as a follow-up test. In fact, measuring serum levels of melatonin among SMS cases whose deletion size is common and has been specified as typical deletion ∼3.7 Mb may be an ideal for these patients. This category represents ∼70% of patients with 17p11.2. In contrast, the limitation of this test may lays in the remaining 30% patients with large, atypical distal 5 Mb deletion of paternal origin, who exhibit normal melatonin rhythm, and then the melatonin test role in these cases is useless.

In conclusion, accurate clinical diagnosis, therapeutic assessments, and management of this unique multisystem disorder in spite of its complications should encompass a holistic management plan including multidiscipline therapeutic strategies, periodic neuro-developmental assessments and/or developmental/behavioral pediatric consultations, and an early intervention programs. However, the uniqueness of the behavioral features of this condition should lead health care providers to request specific FISH testing.

Indeed, beside the medical treatment, which merely relies on managing the symptoms, special education such as vocational training/supports later in life, speech/language intervention, physical, occupational, behavioral, and sensory integration therapies are needed. Respite care and psychosocial support for family members are also highly recommended. Regarding our patients sleep and behavioral disturbances, combinations of the daytime dose of acebutolol (acebutolol 10 mg/kg/day) with an evening oral dose of melatonin (3 mg at 8 pm) alleviate these symptoms noticeably. Currently, no other medications are recommended to him.
