# First Case Report of Smith–Magenis Syndrome (SMS) Among the Arab Community in Nazareth: View and Overview: Erratum

**DOI:** 10.1097/MD.0000000000006673

**Published:** 2017-04-07

In the article “First Case Report of Smith–Magenis Syndrome (SMS) Among the Arab Community in Nazareth: View and Overview”,^[1]^ which appeared in Volume 95, Issue 3 of *Medicine*, Figure 2 was incorrectly included in the article without patient consent. The figure has since been removed from the article.
